# Mid-term outcomes of acetabular fractures treated with acute fix and replace versus ORIF in the elderly: a multicentric study with minimum 5-year follow-up

**DOI:** 10.1007/s00590-025-04309-1

**Published:** 2025-05-14

**Authors:** Amarildo Smakaj, Giuseppe Rovere, Concetto Battiato, Rocco Erasmo, Domenico De Mauro, Andrea Fidanza, Fernando De Maio, Pasquale Farsetti, Francesco Liuzza

**Affiliations:** 1 Policlinico Tor Vergata, Rome, Italy; 2 University of Rome Tor Vergata, Rome, Italy; 3 Catholic University of the Sacred Heart, Milan, Italy; 4 Ospadale Mazzoni, Ascoli Piceno, Italy; 5 Azienda USL di Pescara, Pescara, Italy; 6 Agostino Gemelli University Polyclinic, Rome, Italy; 7 University of Naples Federico II, Naples, Italy; 8 University of L’Aquila, L’Aquila, Italy; 9 University of Rome Tor Vergata, Rome, Italy; 10 Policlinico Tor Vergata, Rome, Italy

**Keywords:** Acetabular fractures, Elderly patients, Total hip arthroplasty, Open reduction and internal fixation, ORIF, Fix and replace, Combined hip procedure, CHP, Mid-term outcomes, Functional results

## Abstract

**Purpose:**

Acetabular fractures in elderly patients are increasing and present complex management challenges. This multicentric retrospective study compares mid-term clinical and radiographic outcomes of acute “fix and replace” versus ORIF, focusing on implant survival, complications, and functional performance at a minimum five-year follow-up, addressing the current lack of extended outcome data.

**Methods:**

This study is an update of a previously published multicentric retrospective cohort including patients aged ≥ 60 years with acetabular fractures treated surgically at three hospitals in central Italy between 2013 and 2025. Patients received either acute “fix and replace” (ORIF combined with acute THA) or ORIF alone, with a minimum clinical and radiographic follow-up of five years. Functional outcomes were assessed using PROMs (SF-12, PDI) and clinician-based scores (HHS, Modified Merle d’Aubigné and Postel). Radiographs were reviewed for healing, implant integrity, osteolysis, loosening, osteoarthritis, and heterotopic ossifications. Complications and implant survival were analyzed using Kaplan–Meier curves with revision surgery as the endpoint.

**Results:**

A total of 41 patients (21 ORIF, 20 CHP) completed a minimum five-year follow-up. No significant differences were found between groups regarding age, BMI, or follow-up duration. Two revisions to total hip arthroplasty occurred in the ORIF group, with no revisions in the CHP group. Radiographic findings, including heterotopic ossifications and implant loosening, were comparable. Functional scores (HHS, MAPM, SF-12 PCS and MCS) showed no significant differences between groups at both two and five years. The CHP group, however, demonstrated significantly lower pelvic discomfort index (PDI) scores at 60 months (*p* = 0.04). No significant intra-group variations were observed over time.

**Conclusion:**

Both ORIF and CHP provide satisfactory mid-term outcomes in elderly patients with acetabular fractures. However, CHP demonstrated fewer reoperations and better pelvic discomfort scores at five years. These findings support the growing evidence favoring acute fix-and-replace strategies in this population.

## Introduction

Acetabular fractures in the elderly represent a growing clinical challenge due to the increasing incidence in an aging population with osteoporotic bone and multiple comorbidities [1, 2]. Over the past 25 years, the incidence of acetabular fractures in patients aged 60 or older has increased 2.4 times in the United States [3]. Open reduction and internal fixation (ORIF) has traditionally been considered the standard approach for managing these injuries. However, ORIF alone has been associated with high rates of fixation failure, conversion to total hip arthroplasty (THA), and unsatisfactory functional outcomes in frail patients [4–6].

Recent studies have demonstrated that acute management with THA, combined with fracture fixation when needed (“fix and replace”), may reduce reoperation rates and improve early mobilization in this population [7, 8]. Moreover, registry-based data and meta-analyses have highlighted lower rates of secondary procedures and better functional scores in elderly patients undergoing acute THA compared to those treated with ORIF alone [9, 10].

Despite encouraging short- and mid-term outcomes with acute “fix and replace” strategies, evidence on mid-term implant survival, complication rates, and functional performance beyond 5 years remains limited. Given the fragility of this patient population and the biomechanical complexity of acetabular fractures, robust data on outcomes at extended follow-up are necessary to guide surgical decision-making. Although patients undergoing ORIF and CHP may differ in clinical presentation, there are scenarios in which both treatment strategies may be considered appropriate. Therefore, comparing outcomes between ORIF and fix-and-replace approaches reflects real-world clinical variability and may help inform individualized treatment planning. 

This multicentric retrospective study aims to compare the mid-term clinical and radiographic outcomes of elderly patients treated with acute “fix and replace” versus ORIF for acetabular fractures, with a minimum follow-up of five years.

## Material and methods

This multicentric retrospective study was conducted at three hospitals located in central Italy between March 2018 and February 2025. The study complied with the Declaration of Helsinki [11], and local Ethics Committees deemed that ethical approval was not required given its retrospective observational nature. The study population included patients aged 60 years or older who sustained acetabular fractures and were treated surgically either with an acute “fix and replace” strategy (open reduction and internal fixation combined with acute total hip arthroplasty) or with open reduction and internal fixation (ORIF) alone. This investigation represents an extension of a previously published cohort [9], with updated data including a minimum clinical and radiographic follow-up of five years.

All patients presenting with displaced acetabular fractures who were deemed eligible for surgery were screened for inclusion. Exclusion criteria were periprosthetic acetabular fractures, pathological fractures, non-operatively treated fractures, and patients who did not complete at least five years of follow-up. Moreover, patients who presented incomplete clinical records or radiological data at the minimum five-year follow-up were excluded from the study. Patients were allocated to treatment groups based on preoperative clinical assessment and fracture characteristics, in accordance with each institution’s protocol and surgeon preference.

Fractures were classified according to the Letournel and Judet system based on preoperative imaging, including standard radiographs and computed tomography scans [9]. The “fix and replace” approach consisted of fracture fixation followed by cementless total hip arthroplasty performed in the same surgical session (Combined Hip Procedure, CHP). In the ORIF group, anatomical reduction and internal fixation were achieved using plates and screws through different approaches, depending on fracture pattern and surgeon preference.

Demographic data including age, sex, body mass index (BMI), American Society of Anesthesiologists (ASA) score, and comorbidities were reviewed [9]. Clinical outcomes were assessed through both patient-reported outcome measures (PROMs) and clinician-administered scoring systems. Quality of life (QoL) was evaluated using the 12-item Short-Form Health Survey (SF-12) and the Pelvic Discomfort Index (PDI) [12]. Clinician-based assessments included the Harris Hip Score (HHS) and the Modified Merle d’Aubigné and Postel score. Radiographic assessment included evaluation of fracture healing, implant integrity, osteolysis, implant loosening according to Engh score, progression to post-traumatic osteoarthritis according to Kellgren and Lawrence and heterotopic ossifications in accordance with Brooker classification [13]. All complications, including dislocations, hardware failure, aseptic loosening, and need for reoperation or conversion to arthroplasty, were documented throughout the follow-up period.

Statistical analysis was conducted using Prism v10.4.1 (GraphPad). Continuous variables were expressed as means with standard deviations and compared between groups using the Student’s t-test or Mann–Whitney U test, based on distribution. Categorical variables were analyzed using the chi-square or Fisher’s exact test. A p-value of less than 0.05 was considered statistically significant. Implant survival analysis was performed using Kaplan–Meier curves, considering any revision surgery as the endpoint.

## Results

**Fig. 1:**
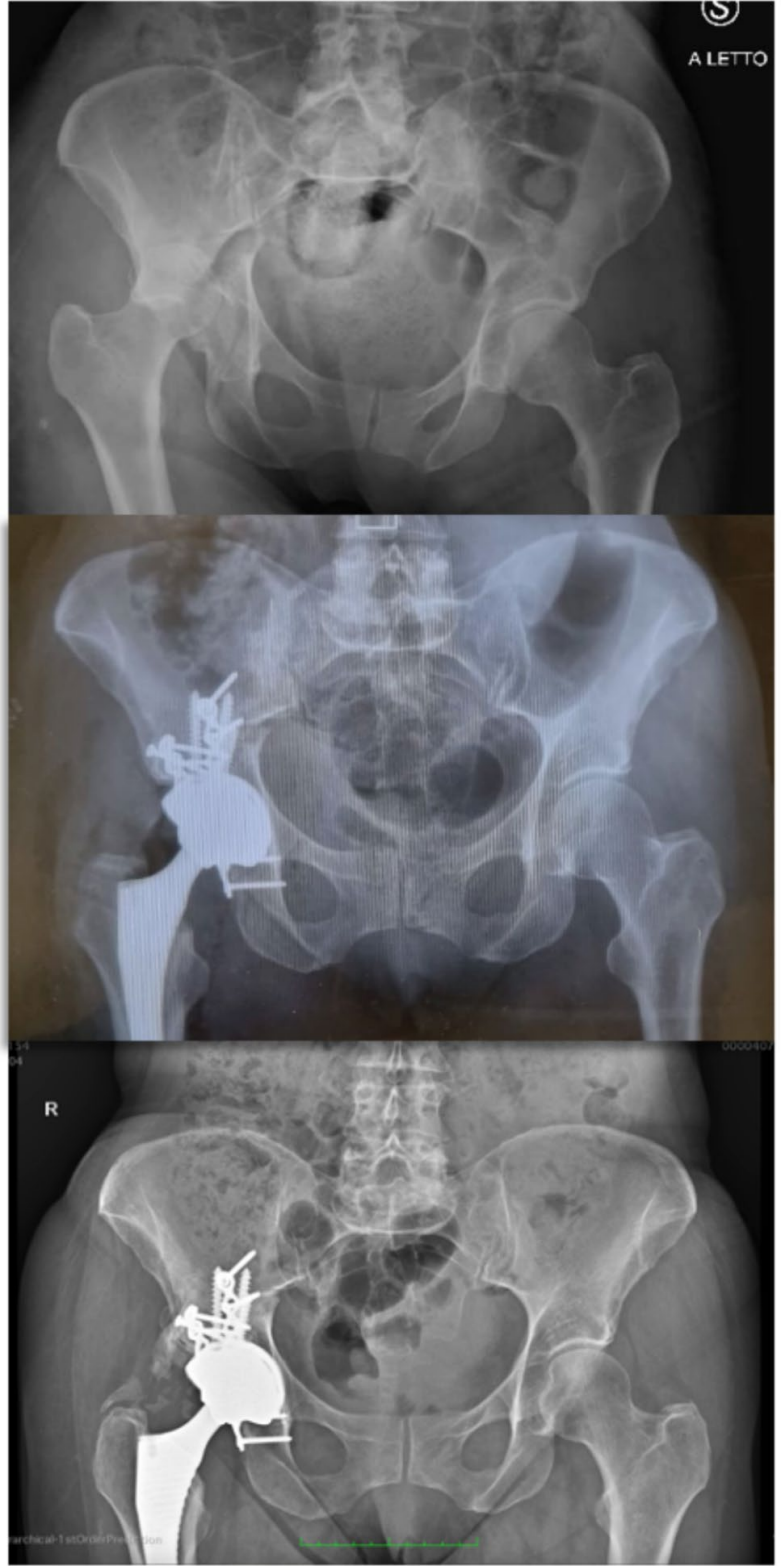
Explicative case of heterotopic ossifications (Brooker 1) in CHP

**Fig. 2:**
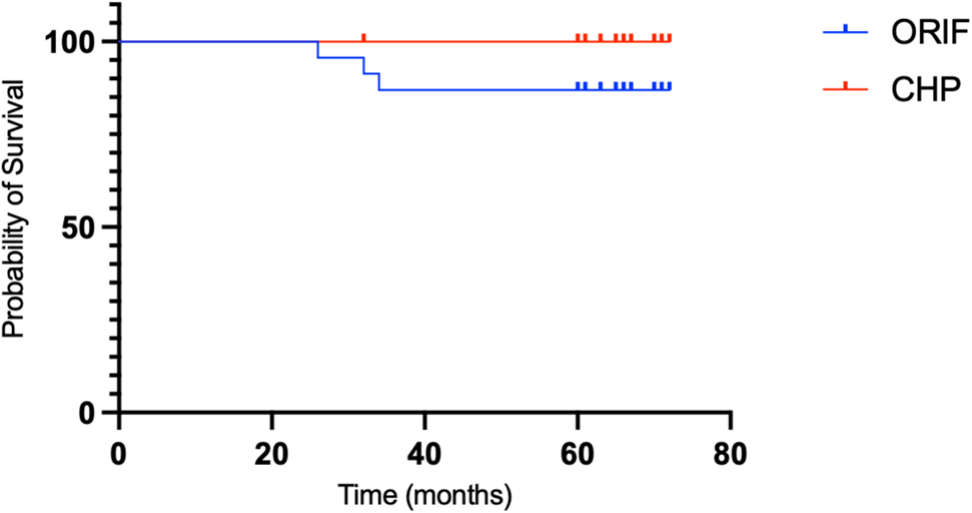
Kaplan–Meier survival analysis illustrating revision-free survival in the ORIF and CHP groups over a minimum follow-up of 5 years

A total of 45 patients were included in the updated cohort, with 24 patients allocated to the CHP group and 21 to the ORIF group. At the minimum five-year follow-up, three patients in the ORIF group and one patient in the CHP group were lost to follow-up and considered as drop-outs. One of the patients of the ORIF group died at 3 years follow-up for heart failure related disease. Therefore, 21 patients in the ORIF group and 20 in the CHP group were available for final analysis. The mean age at the time of surgery was 69.1 ± 5.0 years in the ORIF group and 73.0 ± 8.5 years in the CHP group, with no statistically significant difference between groups (*p* = 0.088). Similarly, there were no significant differences in body mass index (BMI) between the groups. The mean BMI was 24.7 ± 5.3 kg/m^2^ in the ORIF group and 25.5 ± 4.7 kg/m^2^ in the CHP group (*p* = 0.626). The average follow-up duration was 65.4 ± 4.4 months for the ORIF group and 65.0 ± 3.6 months for the CHP group, with no significant difference observed (*p* = 0.705). The demographic data of the patients are summarized in Table 1. At final radiographic evaluation, heterotopic ossifications were identified in both groups. In the ORIF group, 13 patients (61.9%) presented with Brooker grade 1 to 3 ossifications, while in the CHP group, 9 patients (45.0%) showed similar findings. No Brooker grade 4 ossifications were detected in either group (Table 2). An explicative case of CHP is shown in Fig. 1. The distribution of Brooker classifications did not significantly differ between the two cohorts (*p* = 0.89). At the final follow-up, implant loosening was observed in 1 patient (4.2%) in the ORIF group and in 2 patients (9.5%) in the CHP group, all showing radiological signs of loosening without corresponding clinical symptoms. No cases of non-union or septic loosening were observed in either group. Secondary osteoarthritis occurred in 8 patients (29.2%) in the ORIF group. There were two conversions to total hip arthroplasty in the ORIF group within the five-year follow-up period, as illustrated by the Kaplan–Meier survival curve (Fig. 2). Radiographic outcomes and complications are summarized in Table 2. The HHS was comparable between groups at both time points. At 24 months, the ORIF group reported a mean HHS of 79.6 ± 12.4 compared to 83.3 ± 11.1 in the CHP group (*p* = 0.32). At 60 months, values remained similar (78.9 ± 10.7 vs. 83.4 ± 10.4; *p* = 0.18). No significant intra-group variations were noted over time for either group. The PDI showed a non-significant difference at 24 months (53.7 ± 8.3 in ORIF vs. 57.9 ± 6.3 in CHP; *p* = 0.079), but the CHP group displayed a significantly better score at 60 months (58.1 ± 6.2 vs. 53.3 ± 8.1; *p* = 0.04). No significant changes were observed within either group over time. For the MAPM, scores at two years were 8.95 ± 1.93 for ORIF and 8.9 ± 1.74 for CHP ( *p* = 0..93), with comparable values at five years (8.71 ± 1.64 vs. 8.89 ± 1.24; *p* = 0.69). Again, no intra-group differences were detected between 24 and 60 months. The SF-12 physical component (PCS) score was similar between groups both at 24 months (40.8 ± 2.8 in ORIF vs. 41.4 ± 4.0 in CHP; *p * = 0.61) and at 60 months (40.5 ± 3.6 vs. 42.4 ± 2.6; *p* = 0.054). Within each group, PCS scores remained stable over time. For the SF-12 mental component (MCS), no significant differences were observed between ORIF and CHP at either 24 months (40.5 ± 2.2 vs. 41.4 ± 1.9; *p* = 0.15) or 60 months (41.2 ± 1.6 vs. 42.0 ± 1.5; *p* = 0.09). Paired samples t-tests showed no significant variations in MCS scores between two and five years within either treatment group. Table 3 reports functional and quality of life outcomes.

**Table 1:** Demographic and follow-up data of the study population

	ORIF	CHP	*P* value
Number of patients	21	20	–
Male/female Ratio	1:2	1:3	–
Age (years)	69.1 ± 5.0	73.0 ± 8.5	0.09
BMI (kg/m^2^)	24.7 ± 5.3	25.5 ± 4.7	0.63
Follow-up (months)	65.4 ± 4.4	65.0 ± 3.6	0.70

**Table 2:** Radiographic outcomes and complications at final follow-up in the ORIF and CHP groups

	ORIF	CHP	*P* value
Implant loosening*	4.2% (n = 1)	9.5% (n = 2)	1.0
Non-union	–	–	–
Heterotopic ossification**	61.9% (n = 13)	45.0% (n = 9)	0.89
Brooker 1	8	6	
Brooker 2	4	2	
Brooker 3	1	1	
Brooker 4	–	–	
Secondary osteoarthritis	29.2% (n = 8)	/	–
Septic loosening	–	–	–

^*^Engh classification

^**^Brooker classification

**Table 3:** Functional and quality of life outcomes at two and five years for ORIF and fix-and-replace groups

		2y			5 y		
		ORIF	Fix and replace	*P* value	ORIF	Fix and replace	*P* value
SF-12	PCS**	40.8 ± 2.8	41.4 ± 4.0	0.61	40.5 ± 3.6	42.4 ± 2.6	0.054^†^
	MCS^‡^	40.5 ± 2.2	41.4 ± 1.9	0.15	41.2 ± 1.6	42.0 ± 1.5	0.09
Pelvic discomfort index (PDI)		53.7 ± 8.3	57.9 ± 6.3	0.08	53.3 ± 8.1	58.1 ± 6.2	0.04
Harris hip score (HSS)		79.6 ± 12.4	83.3 ± 11.1	0.32	78.9 ± 10.7	83.4 ± 10.4	0.18
MAPM*		8.95 ± 1.9	8.9 ± 1.74	0.93	8.71 ± 1.64	8.89 ± 1.24	0.69

* Modified Merle d’Aubigné and Postel score

^†^ Statistically significant *p* < 0.05

**Physical component score

^‡^Mental component score

## Discussion

Our study provides a comprehensive analysis of mid-term outcomes in elderly patients with acetabular fractures treated either with ORIF alone or CHP. The findings reveal that both treatment modalities yield satisfactory clinical and radiographic results at a minimum follow-up of five years. However, certain distinctions between the groups merit further discussion. Functional assessments were comparable between the ORIF and CHP groups at both two-year and five-year follow-ups. However, the CHP group exhibited a significant improvement in the PDI at the five-year mark (*p* = 0.04), suggesting reduced pelvic discomfort over time. This aligns with existing literature indicating that acute THA can facilitate earlier weight-bearing and potentially enhance patient comfort in the mid term [10, 14]. In particular, Kahhaleh et al., reported that patients over 75 years undergoing CHP had a lower need for revision surgeries and higher ten-year survivorship compared to those treated with ORIF alone [15]. Both studies suggest that CHP may offer advantages in reducing the need for subsequent surgeries in this patient population. However, our study observed comparable functional outcomes between the two groups, whereas Kahhaleh et al. did not report on functional outcomes [15]. Nevertheless, a recent research indicates that aTHA is associated with higher risks of revision, dislocation, and periprosthetic fractures compared to primary THA without a history of acetabular fractures [16]. This discrepancy may be attributed to differences in surgical techniques, patient selection criteria, or postoperative management protocols. In fact, in the study by Alqazzaz et al. patients with acetabular fractures underwent total hip arthroplasty (THA) at varying intervals post-injury and there is no not specified whether fracture fixation was performed prior to or during the THA procedures. This distinction is crucial, as combining fracture fixation with THA may influence outcomes differently compared to THA alone. The study by Lannes et al. [17] compares combined hip procedures (CHP) using dual mobility cups (DM-CHP) to standard ORIF in elderly patients, reporting a reduction in early revision rates and low dislocation rates with DM components [17]. These findings are consistent with our results, which also show fewer conversions to THA in the CHP group compared to ORIF. However, unlike Lannes et al. we did not systematically employ dual mobility cups, which could represent an additional strategy to further reduce postoperative complications in future clinical practice [17].

We observed two revision surgeries in the ORIF group within five years, while no revisions were required in the CHP cohort. Interestingly, despite a 30% incidence of post-traumatic osteoarthritis in the ORIF group, functional scores such as SF-12 and HHS remained comparable to those of the CHP group. This apparent discrepancy may be partially explained by the clinical heterogeneity of post-traumatic osteoarthritis, which does not always correlate with significant symptoms or functional decline. In our cohort, while two patients did progress to conversion THA, others with radiographic signs of joint degeneration likely experienced only mild discomfort, insufficient to influence the assessed outcome measures or to warrant further surgical intervention. The need for conversion to THA following ORIF likely reflects the higher risk of joint degeneration and fixation failure associated with isolated osteosynthesis [18–20]. However, THA after acetabular fracture is associated with significantly lower 10-year survivorship and high complication [21], supporting the growing body of evidence that acute “fix and replace” strategies may reduce the risk of secondary surgical procedures in elderly patients with acetabular fractures [22, 23]. In contrast, the CHP approach addresses both fracture stabilization and joint degeneration from the outset, reducing the likelihood of late mechanical complications requiring revision [17, 24]. Heterotopic ossifications (HO) were identified in both our groups, with a higher incidence observed in the ORIF cohort (61.9%) compared to the CHP group (45.0%), although this difference did not reach statistical significance. The predominance of Brooker grade 1 and 2 ossifications in both groups suggests a relatively mild severity in most cases. The higher occurrence in the ORIF group may be related to prolonged immobilization or a more extensive surgical approach [25, 26]. Interestingly, while the CHP procedure involves additional arthroplasty, which is often considered a risk factor for HO, early mobilization and optimized perioperative management may have mitigated this risk. These findings are in line with previous reports suggesting that early mobilization and weight-bearing protocols can help reduce the incidence of clinically significant HO [27, 28]. Compared to prior literature, our study provides a focused analysis of mid-term outcomes (≥ 5 years) between acute CHP and ORIF in elderly patients with acetabular fractures. Ortega-Briones et al. evaluated combined fixation and THA but with midterm follow-up and without a direct ORIF comparison [29]. Nicol et al. investigated delayed THA following failed ORIF, addressing a salvage scenario rather than an acute CHP strategy [30]. Wilson et al. assessed outcomes between native hips and THA but did not specifically examine the fix-and-replace technique [31]. The AceFIT trial demonstrates the feasibility of a large-scale RCT comparing ORIF, CHP (fix-and-replace), and non-surgical treatment for acetabular fractures in elderly patients [32]. While our study retrospectively compared ORIF and CHP with a mid-term focus, AceFIT highlights the growing interest in prospectively evaluating these strategies.

This study has several strengths. First, it provides one of the few available datasets with a minimum five-year follow-up comparing acute “fix and replace” strategies to ORIF alone in elderly patients with acetabular fractures. The multicentric nature of the study, involving different institutions with diverse surgical teams, enhances the generalizability of our findings to real-world settings. Additionally, multiple validated functional and quality of life outcome measures (HHS, MAPM, PDI, SF-12) were employed, allowing a comprehensive evaluation of both clinical and patient-reported outcomes.However, some limitations should be acknowledged. The retrospective design may introduce selection bias and limits control over confounding variables. Despite a relatively high follow-up rate, a small number of patients were lost to follow-up or excluded due to incomplete data, potentially impacting the study’s power. Furthermore, although statistically comparable, the two groups exhibited a modest age difference at baseline, which could have influenced clinical outcomes. Lastly, the absence of a formal randomization process and the relatively small sample size, inherent to studies focusing on a frail and specific population, may limit the external validity of our conclusions.

## Conclusions

Our findings suggest that both ORIF and acute fix-and-replace (CHP) strategies offer satisfactory mid-term clinical and radiographic outcomes in elderly patients with acetabular fractures. CHP demonstrated a lower reoperation rate and better pelvic discomfort scores at five years, reinforcing its role as a valuable option in this patient population. However, further robust evidence is needed. The ongoing AceFIT trial represents a crucial step toward clarifying treatment indications. In the meantime, studies like ours and additional prospective research with extended follow-up are essential to refine clinical guidelines and optimize outcomes in frail elderly patients.

## Data Availability

No datasets were generated or analysed during the current study.
